# Associations Between Selected Autoimmune and Immune-Mediated Conditions and Lymphoma Among Hospitalized Adults: A National Inpatient Sample Analysis

**DOI:** 10.7759/cureus.116241

**Published:** 2026-09-14

**Authors:** ChiAyrsh Ford, Abdelrazig Suliman, Katherine K Barrerra, Nadin Suleiman, Navjot Kaur

**Affiliations:** 1 Internal Medicine, University of Texas at Tyler Health Science Center, Tyler, USA

**Keywords:** autoimmune diseases, hematologic malignancy, lymphoma, national inpatient sample (nis) and the healthcare cost and utilization project (hcup), rheumatoid arthritis, sjögren's disease, systemic lupus erythematosus

## Abstract

Background: Rheumatoid arthritis (RA), systemic lupus erythematosus (SLE), and Sjögren's disease have been associated with lymphoma, but estimates from large, hospitalized populations remain limited. Asthma is not a classic autoimmune disease and was evaluated as a comparator immune-mediated inflammatory condition.

Objective: To examine the associations of RA, SLE, Sjögren's disease, celiac disease, and asthma with the odds of a recorded diagnosis of lymphoma among adult hospitalizations in the United States.

Methods: A retrospective cross-sectional analysis was conducted using National Inpatient Sample (NIS) discharge records from 2017 to 2020. The analytic sample included 39,838 hospitalizations with lymphoma and a random sample of 39,838 hospitalizations without lymphoma. Separate and mutually adjusted logistic regression models were adjusted for documented tobacco use, HIV infection, organ transplantation, age, race, and sex. A sensitivity analysis excluded hospitalizations involving HIV infection or organ transplantation.

Results: The analysis included 79,676 hospitalizations. In the fully adjusted model, lymphoma was associated with RA, with an adjusted odds ratio (aOR) of 1.30 and a 95% confidence interval (CI) of 1.18-1.45; SLE, with an aOR of 2.12 and a 95% CI of 1.76-2.57; and Sjögren's disease, with an aOR of 2.35 and a 95% CI of 1.79-3.09. All p-values were < 0.001. Celiac disease (aOR: 1.01, 95% CI: 0.71-1.45; p = 0.937) and asthma (aOR: 1.05, 95% CI: 0.98-1.13; p = 0.134) were not independently associated with lymphoma. The sensitivity analysis showed the same overall pattern.

Conclusion: In this balanced inpatient sample, RA, SLE, and Sjögren's disease were independently associated with higher odds of a recorded lymphoma diagnosis, whereas celiac disease and asthma were not independently associated after adjustment. These discharge-level associations do not estimate population prevalence or incidence and should not be interpreted as evidence of causation.

## Introduction

Lymphoma encompasses a heterogeneous group of malignancies arising from the clonal proliferation of lymphocytes. Its pathogenesis is multifactorial, with established associations involving chronic inflammation, infection, impaired immune regulation, and treatment-related immunosuppression [1-5].

Certain autoimmune diseases, particularly those characterized by chronic B-cell activation, germinal center reactions, and autoantibody production, are more strongly associated with lymphoma than others. Hemminki et al. demonstrated that predominantly germinal center-derived B-cell neoplasms, such as diffuse large B-cell lymphoma, follicular lymphoma, and Hodgkin lymphoma, showed the strongest associations with autoimmune diseases, suggesting that autoimmune stimulation interferes with B-cell maturation in the germinal center [6]. This mechanistic framework helps explain why rheumatoid arthritis (RA), systemic lupus erythematosus, and Sjögren's disease, all of which are characterized by germinal center hyperactivity, show stronger lymphoma associations than conditions with different immunopathology [3,6].

RA, SLE, and Sjögren's disease have repeatedly been associated with lymphoma, although the magnitude and subtype distribution differ across conditions [1-5,7,8-12]. Associations reported for celiac disease are less consistent, and asthma is better regarded as an immune-mediated inflammatory comparator than an autoimmune disease. Tobacco smoking has also been investigated as a potential risk factor for lymphoma, with associations varying by lymphoma subtype [13].

Not all autoimmune diseases have shown a consistent association with lymphoma. A Swedish nationwide cohort found no increase in non-Hodgkin lymphoma risk for several conditions, including ankylosing spondylitis, type 1 diabetes, Graves' disease, multiple sclerosis, chorea minor, and pernicious anemia [14]. The InterLymph pooled analysis likewise found no association for several immune-mediated disorders [4], and a later umbrella review reported no increased overall malignancy association with multiple sclerosis [15].

RA, SLE, and Sjögren's disease were selected because their lymphoma associations are supported by a substantial body of epidemiologic and mechanistic evidence. Celiac disease was included because its overall association remains uncertain despite a recognized link with enteropathy-associated T-cell lymphoma [16]. Asthma was included as an immune-mediated inflammatory comparator with predominantly allergic, type 2 inflammatory pathways.

Asthma is a heterogeneous chronic inflammatory airway disease rather than a classic autoimmune disease. Its common allergic phenotypes are characterized predominantly by type 2 inflammation rather than autoantibody-mediated tissue injury [17,18]. Asthma has nevertheless been included in broader studies of immune-mediated diseases and cancer [19]. Therefore, asthma was treated as an immune-mediated inflammatory condition in this study. Although ICD-10-CM includes J45.x subcodes for certain asthma severity categories and clinical states, all J45.x codes were grouped into a single asthma variable in this analysis; therefore, associations according to asthma subtype, severity, or exacerbation status could not be evaluated [20].

The Healthcare Cost and Utilization Project (HCUP) NIS is the largest publicly available all-payer inpatient database in the United States and is well suited to studying associations among diagnoses recorded during hospitalization [21]. This study examined the association of RA, SLE, Sjögren's disease, celiac disease, and asthma with a recorded lymphoma diagnosis.

## Materials and methods

Design and data source 

A retrospective cross-sectional analysis was conducted using the HCUP NIS for 2017-2020. The NIS is a stratified sample of discharge records from US community hospitals and contains demographic, diagnostic, procedural, and hospital-level information. Each record represents one hospitalization rather than one unique patient. Because the NIS does not provide patient identifiers, repeat hospitalizations by the same individual cannot be linked or removed [22].

Analyses were unweighted and did not incorporate NIS discharge weights, discharge strata, or hospital clusters. Accordingly, the findings describe associations within the selected analytic sample and should not be interpreted as nationally representative prevalence or incidence estimates. 

Inclusion and exclusion criteria 

Eligible records represented hospitalizations of adults aged 18-90 years during 2017-2020. Lymphoma was identified from ICD-10-CM diagnoses recorded in any diagnostic field. Records missing variables required for a given regression model were excluded from that model.

Sampling strategy 

The analytic dataset contained 39,838 hospitalizations with lymphoma and an equal-sized random sample of 39,838 hospitalizations without lymphoma. The non-lymphoma records were not matched to lymphoma records on age, sex, year, or other characteristics. Demographic differences were therefore described and addressed through regression adjustment. Because lymphoma status was deliberately balanced, the sample cannot be used to estimate the prevalence of lymphoma among all US hospitalizations.

Study variables

ICD-10-CM coded discharge diagnoses were used to identify hospitalizations with lymphoma and autoimmune/immune-mediated conditions. The ICD-10-CM codes used are presented in Table 1.

**Table 1:** List of ICD-10-CM codes used in the data collection process ICD-10-CM: J45.x International Classification of Diseases, 10th Revision, Clinical Modification (ICD-10-CM) The ICD-10-CM code ranges used to define the study conditions were identified using the National Center for Health Statistics ICD-10-CM Browser Tool [20].

Discharge Diagnosis	ICD-10 Codes
Lymphoma	C81.x (Hodgkin lymphoma), C82.x (Follicular lymphoma), C83.x (Non-follicular lymphoma), C84.x (Mature T/NK-cell lymphomas), C85.x (Other and unspecified types of non-Hodgkin lymphoma), C86.x (Other specified types of T/NK-cell lymphoma)
Rheumatoid Arthritis	M05.x (Seropositive RA), M06.x (Other RA)
Systemic Lupus Erythematosus	M32.x (SLE)
Sjögren's Disease	M35.0x (Sjögren’s syndrome)
Celiac Disease	K90.0 (Celiac disease)
Asthma	J45.x (Asthma)

All diagnoses were identified using ICD-10-CM codes from discharge records, which is the standard approach for NIS-based studies. ICD-coded diagnoses are not equivalent to clinically confirmed diagnoses, and this represents a recognized limitation of administrative database research. For asthma, ICD-10 codes (J45.x) [20] do not distinguish between allergic and non-allergic subtypes or severity levels.

Demographic variables included age, sex, race/ethnicity, and hospital region. The primary outcome was a diagnosis of lymphoma.

Statistical analysis 

Continuous variables were summarized using means and standard deviations, while categorical variables were summarized using counts and percentages. Separate unadjusted binary logistic regression models were used to estimate the associations between the hospital region and each selected condition with lymphoma status. Subsequently, condition-specific adjusted models were fitted for RA, SLE, Sjögren’s disease, celiac disease, and asthma. Each model adjusted for documented current or former tobacco/nicotine use, HIV infection, organ transplantation, age, race, and sex. A fully adjusted model included all five selected conditions and the same covariates simultaneously. Odds ratios (ORs) and aORs are reported with 95% CIs.

As a sensitivity analysis, hospitalizations with HIV infection or a history of organ transplantation were excluded because both conditions can substantially alter lymphoma susceptibility [23,24]. Condition-specific logistic regression models were repeated in the restricted sample with adjustment for age, sex, and documented current or former tobacco/nicotine use. Because the restricted models used a reduced covariate set, they were interpreted as a supportive analysis rather than an exact replication of the fully adjusted primary model.

The ORs describe relative odds within this selected inpatient sample; they do not represent absolute risk, incidence, or population prevalence. Given the cross-sectional discharge-level design, the associations cannot establish a temporal sequence or causation.

All tests were two-sided, and p < 0.05 was considered statistically significant.

## Results

The study included 79,676 adult hospitalizations: 39,838 with lymphoma and 39,838 randomly selected without lymphoma. Hospitalizations with lymphoma predominantly involved older adults on average (67.8 vs 57.8 years), as well as males (54.6% vs 42.6%) and white patients (75.1% vs 65.2%) (Table 2).

**Table 2:** Patient characteristics by lymphoma status Note: Values are n (%) unless otherwise indicated. Percentages use the full lymphoma-status group as the denominator and therefore include missing values. All counts, percentages, and regression estimates are unweighted; NIS discharge weights, strata, and hospital clusters were not applied.

Characteristic	Lymphoma: No (N = 39,838)	Lymphoma: Yes (N = 39,838)
Age at admission, years		
Mean (SD)	57.8 (20.3)	67.8 (16.2)
Median (minimum, maximum)	61.0 (18.0, 90.0)	71.0 (18.0, 90.0)
Sex, n (%)		
Male	16,977 (42.6)	21,742 (54.6)
Female	22,857 (57.4)	18,096 (45.4)
Race, n (%)		
White	25,966 (65.2)	29,914 (75.1)
Black	5,858 (14.7)	3,841 (9.6)
Hispanic	4,362 (10.9)	3,321 (8.3)
Asian or Pacific Islander	1,173 (2.9)	716 (1.8)
Native American	258 (0.6)	136 (0.3)
Other	1,201 (3.0)	1,030 (2.6)
Missing	1,020 (2.6)	880 (2.2)

The demographic distributions differed substantially between groups, confirming that the random comparison sample was not demographically matched. These differences were addressed in the adjusted models rather than interpreted as population prevalence estimates (Table 2).

Hospital region was associated with lymphoma status in the unadjusted analysis (overall p < 0.001). Relative to the northeast, the odds of lymphoma were lower in the midwest (OR: 0.94, 95% CI: 0.90-0.98; p = 0.005), south (OR: 0.96, 95% CI: 0.92-1.00; p = 0.035), and west (OR: 0.91, 95% CI: 0.87-0.95; p < 0.001) (Table 3). These estimates describe regional differences in the selected inpatient sample and should not be interpreted as geographic causes of lymphoma.

**Table 3:** Hospital region by lymphoma status and unadjusted association with lymphoma Note: Values in the lymphoma columns are n (column %). Odds ratios are from unadjusted binary logistic regression with northeast as the reference. All counts, percentages, and regression estimates are unweighted; NIS discharge weights, strata, and hospital clusters were not applied. OR, odds ratio; CI, confidence interval

Hospital region	Lymphoma: No (N = 39,838)	Lymphoma: Yes (N = 39,838)	OR (95% CI)	P-value
Northeast	7,349 (18.4)	7,719 (19.4)	Reference	-
Midwest	8,785 (22.1)	8,673 (21.8)	0.94 (0.90-0.98)	0.005
South	15,907 (39.9)	16,024 (40.2)	0.96 (0.92-1.00)	0.035
West	7,797 (19.6)	7,422 (18.6)	0.91 (0.87-0.95)	<0.001

RA, SLE, and Sjögren's disease were more frequent among lymphoma hospitalizations than among non-lymphoma hospitalizations: 2.6% versus 1.7%, 0.8% versus 0.5%, and 0.5% versus 0.2%, respectively. Celiac disease occurred in 0.2% of each group, while asthma was less frequent among lymphoma hospitalizations (4.5% vs 5.2%) (Table 4). These unadjusted distributions are shown in Figure 1. 

**Table 4:** Prevalence of autoimmune conditions and asthma by lymphoma status Note: Values are n (column %). SLE, systemic lupus erythematosus. All counts, percentages, and regression estimates are unweighted; NIS discharge weights, strata, and hospital clusters were not applied.

Condition	Lymphoma: No (N = 39,838)	Lymphoma: Yes (N = 39,838)
Rheumatoid arthritis		
No	39,157 (98.3)	38,808 (97.4)
Yes	681 (1.7)	1,030 (2.6)
SLE		
No	39,647 (99.5)	39,511 (99.2)
Yes	191 (0.5)	327 (0.8)
Sjögren's disease		
No	39,762 (99.8)	39,634 (99.5)
Yes	76 (0.2)	204 (0.5)
Celiac disease		
No	39,772 (99.8)	39,769 (99.8)
Yes	66 (0.2)	69 (0.2)
Asthma		
No	37,757 (94.8)	38,065 (95.5)
Yes	2,081 (5.2)	1,773 (4.5)

**Figure 1:**
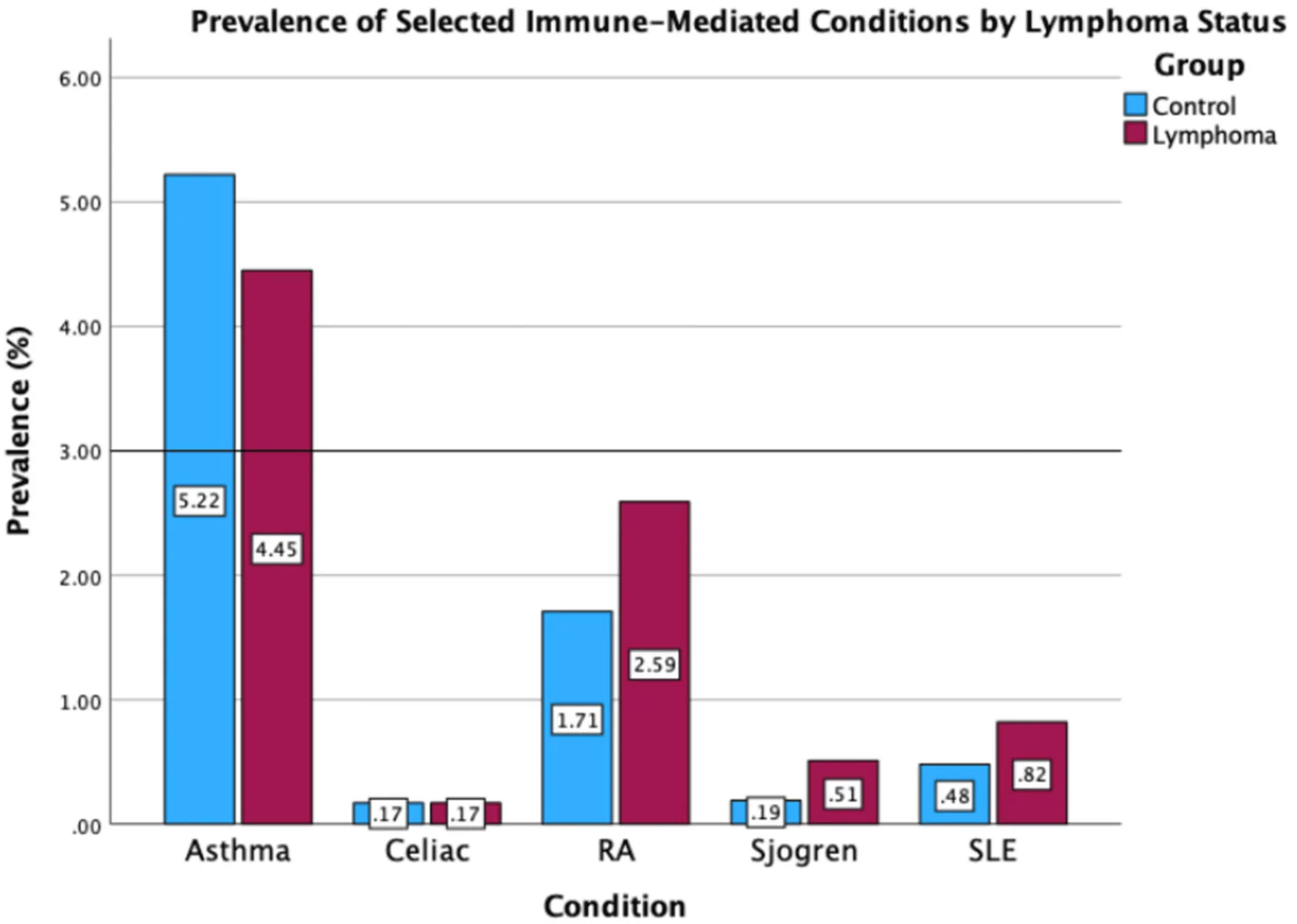
Prevalence of selected immune-mediated conditions among hospitalizations with and without lymphoma Bars show unweighted percentages in the lymphoma and randomly selected comparison groups. These are descriptive prevalence estimates; adjusted associations are reported separately. RA, rheumatoid arthritis; SLE, systemic lupus erythematosus

In unadjusted models, lymphoma was associated with RA (OR: 1.53, 95% CI: 1.38-1.68), SLE (OR: 1.72, 95% CI: 1.44-2.05), and Sjögren's disease (OR: 2.69, 95% CI: 2.07-3.51; all p < 0.001). Celiac disease was not associated with lymphoma (OR: 1.05, 95% CI: 0.75-1.47; p = 0.796). Asthma showed an inverse unadjusted association (OR: 0.85, 95% CI: 0.79-0.90; p < 0.001) (Table 5). 

**Table 5:** Unadjusted associations of autoimmune conditions and asthma with lymphoma Note: Each odds ratio was estimated in a separate unadjusted binary logistic regression model. The reference category was absence of the listed condition. All counts, percentages, and regression estimates are unweighted; NIS discharge weights, strata, and hospital clusters were not applied. OR, odds ratio; CI, confidence interval; SLE, systemic lupus erythematosus

Predictor	OR	95% CI	P-value
Rheumatoid arthritis: Yes	1.53	1.38-1.68	<0.001
SLE: Yes	1.72	1.44-2.05	<0.001
Sjögren's disease: Yes	2.69	2.07-3.51	<0.001
Celiac disease: Yes	1.05	0.75-1.47	0.796
Asthma: Yes	0.85	0.79-0.90	<0.001

The condition-specific adjusted models continued to show higher odds of lymphoma with RA (aOR: 1.39, 95% CI: 1.25-1.54), SLE (aOR: 2.35, 95% CI: 1.94-2.83), and Sjögren's disease (aOR: 2.71, 95% CI: 2.07-3.56; all p < 0.001). Celiac disease (aOR: 1.05, 95% CI: 0.73-1.50; p = 0.796) and asthma (aOR: 0.94, 95% CI: 0.88-1.01; p = 0.102) were not significant (Tables 6-10). 

**Table 6:** Adjusted association of rheumatoid arthritis with lymphoma Note: The rheumatoid arthritis estimate was adjusted for documented tobacco use, HIV, organ transplantation, age, race, and sex. Reference categories were absence of each binary condition, White race, and male sex. The age OR represents a 1-year increase. All counts, percentages, and regression estimates are unweighted; NIS discharge weights, strata, and hospital clusters were not applied. OR, odds ratio; CI, confidence interval

Predictor	Adjusted OR (95% CI)	P-value
Rheumatoid arthritis: Yes	1.39 (1.25-1.54)	<0.001
Documented tobacco use: Yes	0.86 (0.83-0.89)	<0.001
HIV: Yes	6.80 (5.93-7.80)	<0.001
Organ transplant: Yes	1.58 (1.31-1.91)	<0.001
Age, per 1-year increase	1.029 (1.028-1.030)	<0.001
Race		<0.001
White	Reference	-
Black	0.67 (0.64-0.70)	<0.001
Hispanic	0.83 (0.79-0.88)	<0.001
Asian or Pacific Islander	0.62 (0.56-0.69)	<0.001
Native American	0.57 (0.46-0.70)	<0.001
Other	0.87 (0.80-0.95)	0.003
Sex		<0.001
Male	Reference	-
Female	0.66 (0.64-0.68)	<0.001

**Table 7:** Adjusted association of systemic lupus erythematosus (SLE) with lymphoma Note: The SLE estimate was adjusted for documented tobacco use, HIV, organ transplantation, age, race, and sex. Reference categories were absence of each binary condition, White race, and male sex. The age OR represents a 1-year increase. All counts, percentages, and regression estimates are unweighted; NIS discharge weights, strata, and hospital clusters were not applied. OR, odds ratio; CI, confidence interval

Predictor	Adjusted OR (95% CI)	P-value
SLE: Yes	2.35 (1.94-2.83)	<0.001
Documented tobacco use: Yes	0.86 (0.84-0.89)	<0.001
HIV: Yes	6.82 (5.95-7.83)	<0.001
Organ transplant: Yes	1.56 (1.29-1.89)	<0.001
Age, per 1-year increase	1.029 (1.028-1.030)	<0.001
Race		<0.001
White	Reference	-
Black	0.67 (0.64-0.70)	<0.001
Hispanic	0.83 (0.79-0.87)	<0.001
Asian or Pacific Islander	0.62 (0.56-0.68)	<0.001
Native American	0.57 (0.46-0.71)	<0.001
Other	0.87 (0.80-0.95)	0.003
Sex		<0.001
Male	Reference	-
Female	0.66 (0.64-0.68)	<0.001

**Table 8:** Adjusted association of Sjögren's disease with lymphoma Note: The Sjögren's disease estimate was adjusted for documented tobacco use, HIV, organ transplantation, age, race, and sex. Reference categories were absence of each binary condition, White race, and male sex. The age OR represents a 1-year increase. All counts, percentages, and regression estimates are unweighted; NIS discharge weights, strata, and hospital clusters were not applied. OR, odds ratio; CI, confidence interval

Predictor	Adjusted OR (95% CI)	P-value
Sjögren's disease: Yes	2.71 (2.07-3.56)	<0.001
Documented tobacco use: Yes	0.86 (0.84-0.89)	<0.001
HIV: Yes	6.80 (5.92-7.80)	<0.001
Organ transplant: Yes	1.59 (1.31-1.92)	<0.001
Age, per 1-year increase	1.029 (1.028-1.030)	<0.001
Race		<0.001
White	Reference	-
Black	0.67 (0.64-0.70)	<0.001
Hispanic	0.83 (0.79-0.88)	<0.001
Asian or Pacific Islander	0.62 (0.56-0.69)	<0.001
Native American	0.57 (0.46-0.71)	<0.001
Other	0.87 (0.80-0.95)	0.002
Sex		<0.001
Male	Reference	-
Female	0.66 (0.64-0.68)	<0.001

**Table 9:** Adjusted association of celiac disease with lymphoma Note: The celiac disease estimate was adjusted for documented tobacco use, HIV, organ transplantation, age, race, and sex. Reference categories were absence of each binary condition, White race, and male sex. The age OR represents a 1-year increase. All counts, percentages, and regression estimates are unweighted; NIS discharge weights, strata, and hospital clusters were not applied. OR, odds ratio; CI, confidence interval

Predictor	Adjusted OR (95% CI)	P-value
Celiac disease: Yes	1.05 (0.73-1.50)	0.796
Documented tobacco use: Yes	0.86 (0.84-0.89)	<0.001
HIV: Yes	6.79 (5.92-7.79)	<0.001
Organ transplant: Yes	1.58 (1.31-1.91)	<0.001
Age, per 1-year increase	1.029 (1.028-1.030)	<0.001
Race		<0.001
White	Reference	-
Black	0.67 (0.64-0.70)	<0.001
Hispanic	0.83 (0.79-0.87)	<0.001
Asian or Pacific Islander	0.62 (0.56-0.68)	<0.001
Native American	0.57 (0.46-0.71)	<0.001
Other	0.87 (0.80-0.95)	0.003
Sex		<0.001
Male	Reference	-
Female	0.67 (0.65-0.69)	<0.001

**Table 10:** Adjusted association of asthma with lymphoma Note: The asthma estimate was adjusted for documented tobacco use, HIV, organ transplantation, age, race, and sex. Reference categories were absence of each binary condition, White race, and male sex. The age OR represents a 1-year increase. All counts, percentages, and regression estimates are unweighted; NIS discharge weights, strata, and hospital clusters were not applied. OR, odds ratio; CI, confidence interval

Predictor	Adjusted OR (95% CI)	P-value
Asthma: Yes	0.94 (0.88-1.01)	0.102
Documented tobacco use: Yes	0.86 (0.84-0.89)	<0.001
HIV: Yes	6.78 (5.91-7.78)	<0.001
Organ transplant: Yes	1.58 (1.31-1.91)	<0.001
Age, per 1-year increase	1.029 (1.028-1.030)	<0.001
Race		<0.001
White	Reference	-
Black	0.67 (0.64-0.70)	<0.001
Hispanic	0.83 (0.79-0.87)	<0.001
Asian or Pacific Islander	0.62 (0.56-0.68)	<0.001
Native American	0.57 (0.46-0.71)	<0.001
Other	0.87 (0.80-0.95)	0.003
Sex		<0.001
Male	Reference	-
Female	0.67 (0.65-0.69)	<0.001

When all five conditions were included together with documented tobacco use, HIV infection, organ transplantation, age, race, and sex, the study found that RA, SLE, and Sjögren's disease remained significantly associated with lymphoma, whereas celiac disease and asthma did not (Table 11). The results were similar after excluding hospitalizations with HIV infection or organ transplantation, with RA, SLE, and Sjögren's disease remaining associated with lymphoma, while celiac disease and asthma did not show significant associations (Appendix, Supplementary Table S1). The mutually adjusted estimates are summarized in Figure 2.

**Table 11:** Fully adjusted associations with lymphoma Note: All estimates were mutually adjusted for rheumatoid arthritis, SLE, Sjögren's disease, celiac disease, asthma, documented tobacco use, HIV, organ transplantation, age, race, and sex. Reference categories were absence of each binary condition, White race, and male sex. The age OR represents a 1-year increase. All counts, percentages, and regression estimates are unweighted; NIS discharge weights, strata, and hospital clusters were not applied. OR, odds ratio; CI, confidence interval

Predictor	Adjusted OR (95% CI)	P-value
Rheumatoid arthritis: Yes	1.30 (1.18-1.45)	<0.001
SLE: Yes	2.12 (1.76-2.57)	<0.001
Sjögren's disease: Yes	2.35 (1.79-3.09)	<0.001
Celiac disease: Yes	1.01 (0.71-1.45)	0.937
Asthma: Yes	1.05 (0.98-1.13)	0.134
Documented tobacco use: Yes	0.86 (0.83-0.89)	<0.001
HIV: Yes	6.82 (5.95-7.83)	<0.001
Organ transplant: Yes	1.57 (1.30-1.89)	<0.001
Age, per 1-year increase	1.029 (1.028-1.030)	<0.001
Race		<0.001
White	Reference	-
Black	0.67 (0.64-0.70)	<0.001
Hispanic	0.83 (0.79-0.87)	<0.001
Asian or Pacific Islander	0.62 (0.56-0.69)	<0.001
Native American	0.57 (0.46-0.70)	<0.001
Other	0.87 (0.80-0.95)	0.002
Sex		<0.001
Male	Reference	-
Female	0.66 (0.64-0.68)	<0.001

**Figure 2:**
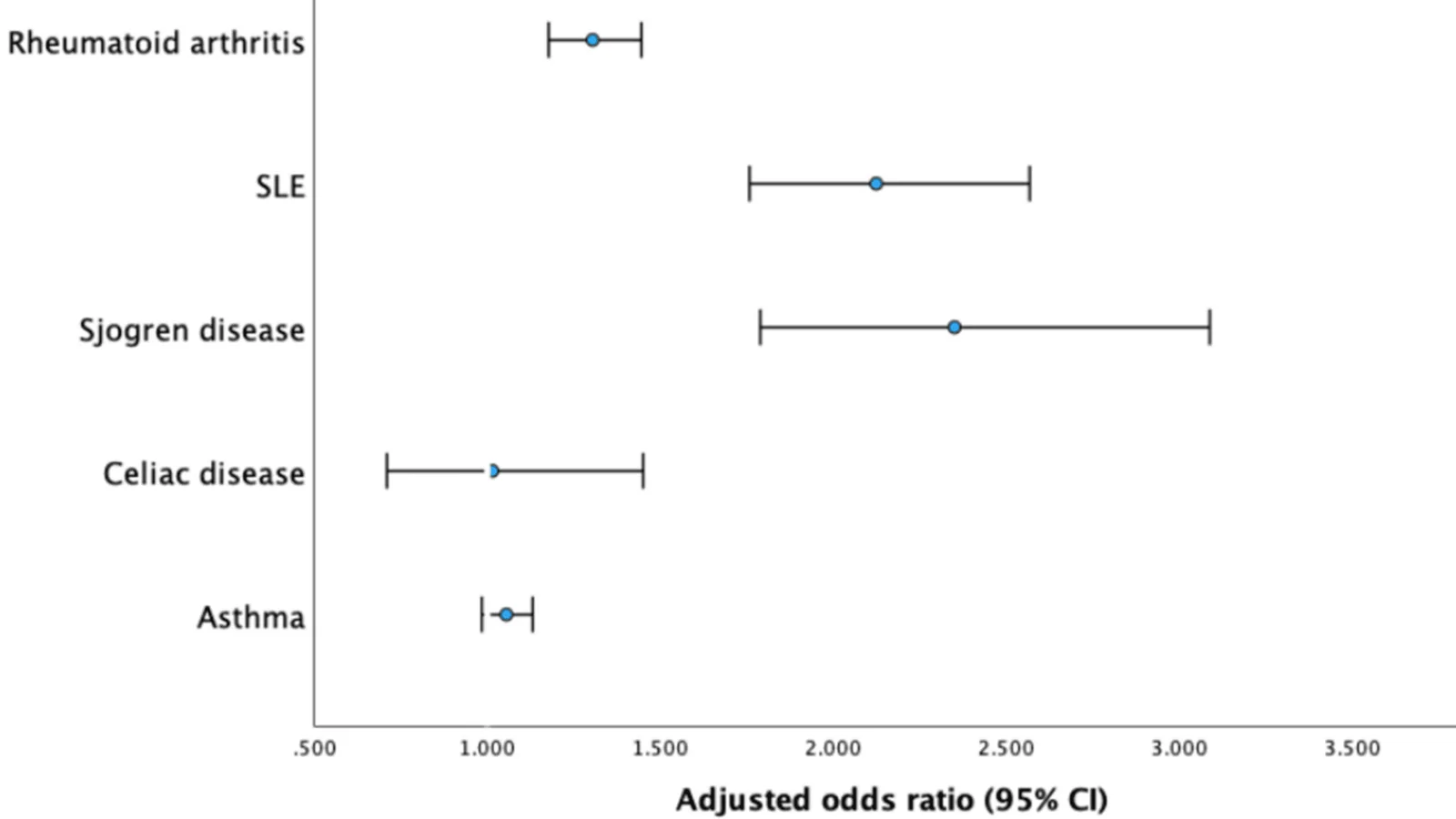
Fully adjusted associations of selected autoimmune and immune-mediated conditions with lymphoma Note: Points represent adjusted odds ratios and horizontal bars represent 95% confidence intervals. The unweighted model included rheumatoid arthritis, systemic lupus erythematosus, Sjögren's disease, celiac disease, asthma, documented tobacco use, HIV infection, organ transplantation, age, sex, and race. The vertical line denotes the null value (OR = 1.0).

## Discussion

The fully adjusted model showed independent associations of lymphoma with RA (aOR: 1.30, 95% CI: 1.18-1.45), SLE (aOR: 2.12, 95% CI: 1.76-2.57), and Sjögren's disease (aOR: 2.35, 95% CI: 1.79-3.09; all p < 0.001). Celiac disease (aOR: 1.01, 95% CI: 0.71-1.45; p = 0.937) and asthma (aOR: 1.05, 95% CI: 0.98-1.13; p = 0.134) were not independently associated with lymphoma (Table 11).

These findings are consistent with earlier studies. Persistent inflammatory activity has been associated with lymphoma in RA, national data have shown an association between SLE and lymphoma, and lymphoma is a recognized complication of Sjögren's disease, particularly mucosa-associated lymphoid tissue lymphoma [1,8-10,25].

An important consideration when interpreting these findings is the potential confounding effect of immunosuppressive therapy. A recent large-scale cohort study from the Longevity Improvement & Fair Evidence (LIFE) study demonstrated that, among immunosuppressive and immunomodulatory agents, calcineurin inhibitors, iguratimod, and methotrexate were each independently associated with a higher risk of lymphoma, and use of two or more medications was associated with a substantially higher risk (HR: 2.7) [26]. Similarly, Smedby et al. reported that the use of nonsteroidal anti-inflammatory drugs (NSAIDs), systemic corticosteroids, and selected immunosuppressants were associated with non-Hodgkin lymphoma risk in patients with RA. However, multivariable adjustment for treatment had little effect on the risk estimates, suggesting that disease activity itself may be the primary driver [27]. The NIS database does not contain information on outpatient medication use, disease duration, or treatment history, which is a recognized limitation of administrative databases [25,26]. Large pooled studies, including the InterLymph Consortium, have shown that association with lymphoma differs across autoimmune conditions and lymphoma subtypes [4]. These findings support a role for prolonged immune stimulation and dysregulation in lymphomagenesis [5].

Lymphoma is heterogeneous and includes Hodgkin lymphoma and several non-Hodgkin lymphoma subtypes [14]. Different autoimmune diseases may be associated with different subtypes. For example, Sjögren's disease has a particularly strong association with marginal zone and mucosa-associated lymphoid tissue lymphomas, whereas RA and SLE have more often been linked with diffuse large B-cell lymphoma [28]. The present analysis combined lymphoma codes and therefore cannot provide subtype-specific estimates.

This study did not find a significant association between celiac disease and lymphoma. This is consistent with recent research showing that, although certain types of lymphoma, particularly enteropathy-associated T-cell lymphoma, may be more common in people with celiac disease, the overall risk is variable and may differ across populations and with disease severity [9].

The association between asthma and lymphoma changed after adjustment. Although asthma was associated with lower odds of lymphoma in the unadjusted model, the estimate moved toward the null and was no longer statistically significant after it was adjusted. In the fully adjusted model, the point estimate was slightly above 1.0 but remained nonsignificant. Therefore, these data do not support describing asthma as protective against lymphoma.

Several factors may have contributed to this finding. Confounding by indication is possible, as patients hospitalized with asthma may differ systematically from other hospitalized patients in ways that affect the likelihood of a concurrent lymphoma diagnosis, including younger age and different comorbidity profiles. Coding and ascertainment bias may also have contributed. Asthma is common and may be consistently coded across hospitalizations, whereas lymphoma coding may be more frequently coded during oncology-related admissions, potentially resulting in differential ascertainment. Selection bias is another possibility, as hospitalized patients with asthma may not be representative of the broader asthma population, and the reasons for hospitalization may affect the likelihood of a concurrent lymphoma diagnosis. Finally, competing immune pathways may play a role. The Th2-dominant immune response in allergic asthma could theoretically influence immunosurveillance, although this explanation remains speculative, and the available evidence is limited [29].

Previous studies examining atopy and lymphoma have reported mixed findings. An InterLymph pooled analysis found a modest inverse association between atopic disease and non-Hodgkin lymphoma, but asthma alone was not independently associated with lymphoma after accounting for other atopic conditions [30]. Differences in age, comorbidity, healthcare use, diagnostic coding, and selection into the inpatient sample may account for the unadjusted inverse association observed in this study. The adjusted findings are more consistent with no clear independent association between asthma and lymphoma.

Older age, male sex, HIV infection, and organ transplantation were strongly associated with lymphoma in the adjusted models. Race was also associated with lymphoma status, although these differences may reflect a combination of disease epidemiology, healthcare access, diagnostic practices, and the composition of the selected inpatient sample rather than a single biological mechanism (Tables 6-11).

Regional findings were unadjusted and should therefore be interpreted cautiously. The balanced analytic sample was not matched within regions and may not represent the broader hospitalized population in each region.

From a clinical perspective, clinicians caring for patients with RA, SLE, and Sjögren's disease should be aware of the higher odds of lymphoma. Unexplained lymphadenopathy, constitutional symptoms, or cytopenias in these patients should prompt consideration of a possible lymphoproliferative disease [31]. 

Limitations

This study has several important limitations inherent to the use of administrative databases:

Medication and treatment data: The NIS does not capture outpatient medication use, disease duration, or treatment history. Immunosuppressive therapy is an important potential confounder in the relationship between autoimmune diseases and lymphoma. Future studies using databases with medication exposure data, such as claims-based longitudinal databases, are needed to better distinguish the contributions of disease activity from treatment-related immunosuppression.

Disease duration and severity: The NIS does not capture disease duration, severity indices such as Disease Activity Score-28 (DAS28) for RA or Systemic Lupus Erythematosus Disease Activity Index (SLEDAI) for SLE, or disease activity at the time of hospitalization. Disease duration and severity of inflammatory activity may influence lymphoma risk.

ICD coding misclassification: Administrative databases rely on ICD-coded diagnoses, which are subject to coding errors, under-coding, and misclassification. The accuracy of ICD-10 codes for autoimmune diseases in administrative databases varies, and some conditions may be under-reported when they are not the primary reason for hospitalization.

Multiple hospitalizations: The NIS is a discharge-level database without unique patient identifiers (post-2011), so the same patient may account for more than one hospitalization. This may result in correlated observations and could affect the estimated associations, particularly among patients with chronic autoimmune diseases or lymphoma.

Residual confounding: Tobacco use, HIV infection, organ transplantation, age, race, and sex were included in the updated models. However, immunosuppressive medication exposure, autoimmune disease activity and duration, obesity, Epstein-Barr virus infection, socioeconomic factors, and other clinical characteristics were unavailable or incompletely captured and may still contribute to residual confounding.

Lymphoma subtype analysis: The ICD-10 codes used in this study did not allow for detailed analysis of specific lymphoma subtypes. This is an important limitation because different autoimmune diseases may be associated with different lymphoma subtypes.

Cross-sectional design: The NIS provides a cross-sectional snapshot of hospitalizations and does not allow for longitudinal follow-up or determination of disease incidence. Therefore, causal inferences cannot be drawn from this study.

Outcome-based sampling: The analytic dataset was deliberately balanced to include equal numbers of lymphoma and non-lymphoma hospitalizations. Therefore, this study cannot estimate the prevalence or incidence of lymphoma in the NIS population, and the reported ORs describe associations within the selected sample.

Complex survey design: The analyses did not incorporate NIS discharge weights, strata, or hospital clusters. Therefore, the estimates are not nationally representative, and the standard errors do not account for the complex sampling design of the NIS. 

Asthma classification: ICD-10 codes for asthma (J45.x) do not distinguish between allergic and non-allergic subtypes or different levels of severity. Therefore, the analysis could not assess whether the association with lymphoma differed by asthma phenotype or severity.

## Conclusions

In this balanced sample of adult hospitalizations, RA, SLE, and Sjögren's disease were independently associated with higher odds of a recorded lymphoma diagnosis after accounting for measured demographic and clinical covariates. Celiac disease and asthma were not independently associated with lymphoma. The unadjusted inverse association between asthma and lymphoma was attenuated after adjustment and should not be interpreted as evidence of a protective effect. After excluding hospitalizations with HIV infection or organ transplantation, the associations with RA (aOR: 1.41, 95% CI: 1.27-1.56), SLE (aOR: 2.23, 95% CI: 1.86-2.68), and Sjögren's disease (aOR: 2.82, 95% CI: 2.15-3.69) remained significant. Celiac disease remained unassociated with lymphoma, while asthma remained borderline but did not reach statistical significance (aOR: 1.07, 95% CI: 1.00-1.15; p = 0.051).

These results support continued investigation of lymphoma associations in RA, SLE, and Sjögren's disease, but they do not establish individual risk or justify a national screening program. Prospective studies with longitudinal follow-up, medication exposure, disease activity, and lymphoma subtype information are needed before these findings can inform clinical risk-stratification strategies. Future studies should also use population-based designs that preserve the natural frequency of lymphoma and apply comparable covariate structures across primary and sensitivity analyses.

## Appendices

 *Supplementary Table S1*

**Table 12:** Sensitivity analysis excluding patients with HIV infection or organ transplantation Note: Primary estimates are from the fully adjusted model that included all five selected conditions, documented tobacco use, HIV infection, organ transplantation, age, race, and sex. Sensitivity analyses excluded hospitalizations with HIV infection or organ transplantation and used condition-specific models adjusted for age, sex, and documented tobacco use. Because the covariate structures differed, the sensitivity results should be interpreted as supportive rather than as a direct like-for-like replication. All counts, percentages, and regression estimates are unweighted; NIS discharge weights, strata, and hospital clusters were not applied. aOR, adjusted odds ratio; CI, confidence interval

Condition	Primary analysis aOR (95% CI)	Sensitivity analysis aOR (95% CI)	Sensitivity P-value
Rheumatoid arthritis	1.30 (1.18-1.45)	1.41 (1.27-1.56)	<0.001
Systemic lupus erythematosus	2.12 (1.76-2.57)	2.23 (1.86-2.68)	<0.001
Sjögren's disease	2.35 (1.79-3.09)	2.82 (2.15-3.69)	<0.001
Celiac disease	1.01 (0.71-1.45)	1.08 (0.76-1.54)	0.661
Asthma	1.05 (0.98-1.13)	1.07 (1.00-1.15)	0.051
